# Conjugated Polymers Containing EDOT Units as Novel Materials for Electrochromic and Resistance Memory Devices

**DOI:** 10.3390/polym14224965

**Published:** 2022-11-16

**Authors:** Zipeng He, Haoran Xu, Yuhang Zhang, Yanjun Hou, Haijun Niu

**Affiliations:** 1 Key Laboratory of Chemical Engineering Process and Technology for High-Efficiency Conversion, College of Heilongjiang Province, Heilongjiang University, Harbin 150080, China; 2 Key Laboratory of Functional Inorganic Material Chemistry, Ministry of Education of the People’s Republic of China, Heilongjiang University, Harbin 150080, China

**Keywords:** electrochromic, resistance memory, EDOT

## Abstract

Four new multifunctional polymers (P1, P2, P3, and P4) containing EDOT units were synthesized by the Stille coupling reaction. A PL spectrum test found that the introduction of large conjugated groups led to strong fluorescence emission in all polymers. Among the electrochromic and switch properties, these polymer films exhibited reversible color changes, as well as good coloring efficiency. Electrochemical experiments found that the initial oxidation potentials of the polymers were 1.27 V, 1.67 V, 1.78 V, and 1.58 V, respectively. Among them, P3 showed a higher coloring efficiency (283.3 cm^2^·C^−1^), and P2 showed a shorter response time (t_c_ = 2.2 s, t_b_ = 2.8 s). These polymers all exhibited electrochromic and resistive switching storage characteristics. They have good solubility in many organic solvents. In the resistance switch memory characteristics, all polymers had obvious memristive properties, and P4 exhibited a larger switch current ratio (2527.42) and a smaller threshold voltage (0.9 V).

## 1. Introduction

Certain materials undergo stable and reversible changes in optical properties, such as color or transmittance, when subjected to an applied electric field. This phenomenon is called electrochromism (EC) [1,2]. In recent years, materials with electrochromic properties have gradually become a research hotspot [3,4,5]. Currently, electrochromic devices (ECDs) are widely used in smart windows, displays, wearable devices, vision and smartphone detection, electrochromic sensors, electrochromic supercapacitors, and other fields [6,7]. EC materials have the advantages of vivid color changes under light, low drive voltage, and controllable color rendering [8]. However, many electrochromic polymers exhibit aggregation-caused quenching (ACQ) [9,10,11]; they are not appropriate for dim environment applications, which greatly limits their application in ECDs. Therefore, we tried to eliminate the ACQ effect to obtain a high-efficiency fluorescent-conjugated polymer and broaden the scope of fluorescent display. EC materials are also candidate electroluminescent materials [12]. The ideal fluorescent electrochromic polymer should have two parts: one is the twisted structure, and the other is the huge rigidity. We tried to adjust the stacking distance of π-π* by introducing large conjugated elements to prevent the interaction between molecules [13].

Organic electrical memory devices (ORRAMs) have broad application prospects. The active layer of traditional ORRAMs is mainly small organic molecules, but the interaction between small organic molecules is weak. Under different voltages, an ORRAM can realize a reversible change in dynamic resistance. Because the structure and principle of memristors and electrochromic devices are quite different, there are few reports on this kind of dual-functional materials. This combination can bring new applications to future electronic information technology. For instance, the resistance state of the device can be directly observed through color change. Some disadvantages of devices fabricated using organic small molecules, such as high-threshold voltage, poor long-term stability, and low thermal stability, limit their potential application prospects [14]. However, because of their simple processing, low cost, good stability, and low power consumption, high-molecular-weight materials have become a hot research topic.

Polythiophene has excellent photoelectric properties and electrical stability. The conjugated polymers of 3,4-ethyldiothiophene (EDOT) and thienyl groups are typical polythiophene compounds, and they all have significant electron donor properties [15]. In particular, polymers with an EDOT structure have outstanding advantages in photoelectric properties [16,17], so adding EDOT units to the process of polymer synthesis has gradually become a hot spot [18,19].

In this work, we first synthesized four different dibromo monomers and then polymerized them with EDOT to obtain four different multifunctional polymers, which were coded as P1, P2, P3, and P4. The structures of the polymers were confirmed with ^1^H NMR, ^13^C NMR, and FT-IR. Because the combination of EDOT and a large-area-conjugated structure can effectively promote the electron transport of polymers, these polymers have electrochromic-, fluorescence-, and resistance-switching memory characteristics. We studied their thermal-, solubility-, optical-, electrochromic-, and resistance-switching memory properties. The results show that the new polymers have broad application prospects in realizing high-efficiency, high-performance, low-cost ECD and resistive memory storage devices.

## 2. Experimental Section

### 2.1. Materials

3,4-ethylenedioxythiophene (EDOT) (99%), 2,6-dibromoanthraquinone (96%), 4-bromobiphenyl (98%), chrysene (98%), anthraquinone (97%), o-phenylenediamine (98%), thionyl chloride (SOCl_2_) (99%), hydrobromic acid (40%), n-butyllithium solution (2.5 M), tributyltin chloride (96%), N-methyl-2-pyrrolidone (NMP) (99%), methanol (AR), and petroleum ether (AR) were bought from Aladdin Reagent Co. Ltd. Tetrabutylammonium perchlorate (TBAP), N,N-dimethylformamide (DMF) (AR) and tetrahydrofuran (THF) (AR) were purchased from Beijing Chemical Factory (Beijing, China).

### 2.2. Characterization

^1^H NMR and ^13^C NMR were obtained on a Bruker AC 400 MHz spectrometer (which comes from Bruker, Karlsruhe, Germany) with a resonance frequency of 400 MHz and a solvent of deuterated CHCl_3_. FT-IR spectra were obtained using a Model 100 PerkinElmer spectrophotometer (which comes from PerkinElmer, Waltham, MA, USA), and ultraviolet–visible (UV-vis) spectra were obtained using a Shimadzu UV-2501 spectrophotometer(which comes from Shimadzu, Kyoto, Japan). Thermogravimetric analysis (TGA) was obtained using a PerkinElmer-Pyris-6 thermogravimetric analyzer (which comes from PerkinElmer, Waltham, MA, USA). Molecular weight was measured using the Viscotek-VE3580-RI-DETECTOR gel chromatograph. Density-functional theory (DFT) studies were carried out with Gaussian 03 software using Becke’s three-parameter gradient-corrected functional (B3LYP) on an SGI Origin 350 server. The surface roughness was measured using an SHL3-NanoFirst-2000-AFM (Tianjin Huike Instrument Equipment Co., LTD, Tianjin, China). Cyclic voltammetry was measured using a CHI 660A electrochemical workstation (Shanghai Chenhua Instrument Co., Ltd., Shanghai, China). Ag/AgCl and KCl solutions (saturated) were used as reference electrodes, and Pt electrodes were used as counter electrodes. Electrochemical and kinetic studies were performed using an electrochemical workstation coupled with UV-vis spectroscopy. The current−voltage characteristics of the resistance-switching memory device were tested by a Keithley 4200 semiconductor parameter tester (Guangzhou Meidak Data Technology Co., Ltd., Guangzhou, China).

### 2.3. Synthesis of the Compounds

A total of four monomers and four polymers were synthesized. The molecular structures of the monomers were characterized by ^1^H NMR, and the structures of the polymers were characterized by ^1^H NMR, ^13^C NMR, and FT-IR. Among them, ^1^H NMR and ^13^C NMR are summarized in the auxiliary information (Figures S1–S10).

**Scheme 1:**
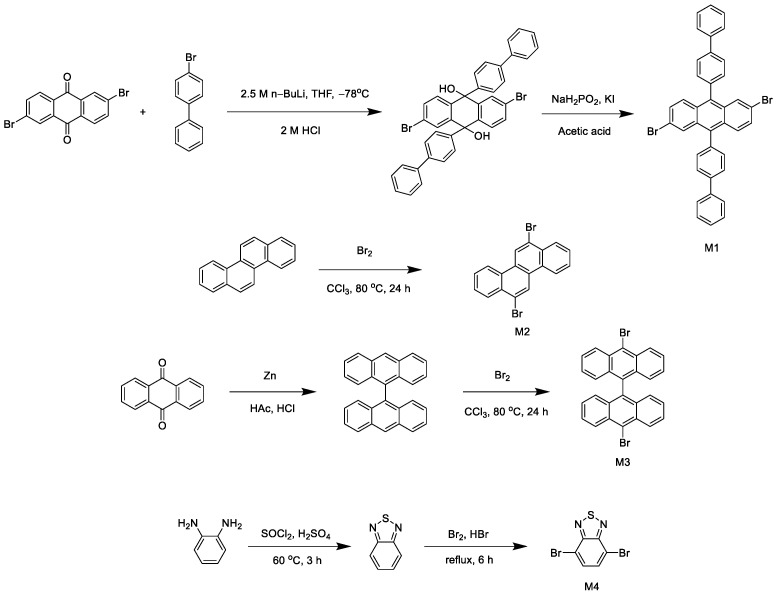

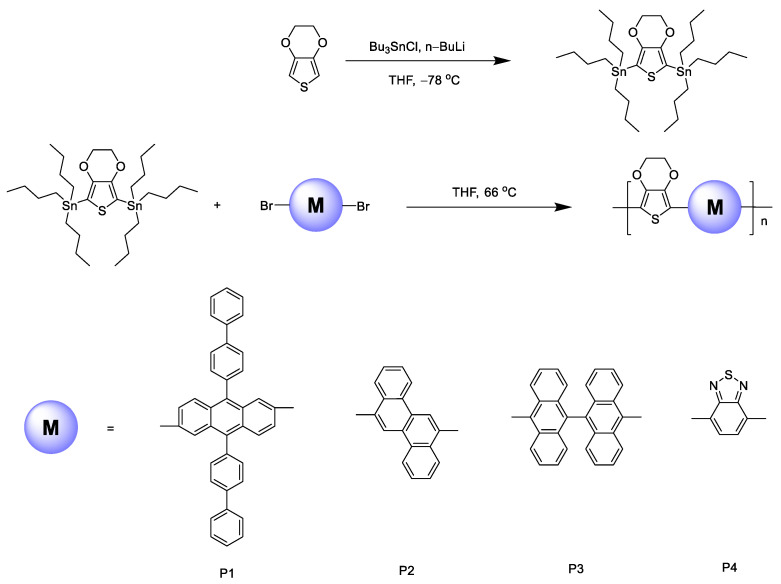
Synthesis routes of the monomers and polymers.

Scheme 1 shows the synthetic routes of the four monomers and polymers.

#### 2.3.1. Synthesis of M1

According to methods from the literature [20,21], 2,6-dibromoanthraquinone (0.37 g, 1 mmol) was added to ethyl ether (50 mL), and the solution was cooled to −78 °C; then, a N_2_ atmosphere was maintained, and n-BuLi (2.5 M, 0.8 mL) was added to the solution. After stirring at −78 °C for 1 h, 4-bromobiphenyl (0.51 g, 2.2 mmol) was added and reacted at −78 °C for 1 h. The solution was poured into the aqueous HCl solution (2 M), the intermediate product was extracted, and then we directly mixed the intermediate with NaH_2_PO_2_ (0.53 g, 5 mmol) and KI in acetic acid (100 mL). After stirring for 3 h, the precipitation produced was filtered and washed repeatedly with water to obtain 2.56 g of yellow solid with a yield of 40%. ^1^H NMR (400 MHz, chloroform-*d*) δ 7.92 (d, *J* = 1.9 Hz, 2H), 7.87 (d, *J* = 8.0 Hz, 4H), 7.82 − 7.76 (m, 4H), 7.65 (d, *J* = 9.4 Hz, 2H), 7.59 − 7.49 (m, 8H), and 7.47 − 7.39 (m, 4H).

#### 2.3.2. Synthesis of M2

According to the method from the literature [22], chrysene (0.23 g, 1 mmol) and 50 mL of chloroform were added to a 100 mL single-necked flask and mixed well; liquid bromine (0.80 g, 5 mmol) was slowly added dropwise, heated to 80 °C, and reacted for 24 h. After the reaction, the mixture was poured into 500 mL of water, and solids were precipitated out, which were filtered and dried to obtain 0.32 g of a white solid product with a yield of 84%. ^1^H NMR (400 MHz, chloroform-*d*) δ 9.01 (s, 2H), 8.77 − 8.65 (m, 2H), 8.50 − 8.38 (m, 2H), and 7.77 (tt, *J* = 7.5, 5.6 Hz, 4H).

#### 2.3.3. Synthesis of M3

According to methods from the literature [23,24], 60 mL of glacial acetic acid, anthraquinone (2.5 g, 12 mmol), and 5.5 g of zinc powder were added to a three-necked flask, which was filled with nitrogen, heated, and stirred. We slowly added 15 mL of concentrated hydrochloric acid dropwise while keeping the temperature at 80–90 °C. The reaction was then carried out at 90 °C for 8 h; the mixture became darker, and the solid gradually precipitated. The solids were filtered and washed with toluene. After drying, 1.7 g of the product was obtained in an 80% yield. We added 10,10′-dibromo-9,9′-bianthracene (0.35 g, 1 mmol) and 50 mL of chloroform to a 100 mL single-necked flask, mixed it well, slowly added bromine (0.80 g, 5 mmol), and heated it at 80 °C for 24 h. After the reaction, the mixture was poured into 500 mL of water, and the solid was precipitated, filtered, and dried; 0.4 g of a brown solid product was obtained in a 78% yield. ^1^H NMR (400 MHz, Chloroform-*d*) δ 8.71 (d, *J* = 8.9 Hz, 4H), 7.58 (dd, *J* = 9.0, 6.4 Hz, 4H), and 7.23 − 6.97 (m, 8H).

#### 2.3.4. Synthesis of M4

According to the method from the literature [25], O-phenylenediamine (10.8 g, 0.1 mol) and SOCl_2_ (100 mL) were added to the flask and stirred thoroughly; then, 1 mL of sulfuric acid was slowly dripped, stirred at 60 °C, and reacted for 3 h. Then, the mixture was slowly mixed and poured into 300 mL of ice water, and it was precipitated and filtered. The crude product was purified into a white solid via column chromatography. The eluent was petroleum ether/ethyl acetate; the yield was 90%. The white solid (6.8 g, 0.5 mol) was dissolved in hydrobromic acid (40%, 90 mL), and then a hydrobromic acid (40%, 100 mL) solution of Br_2_ (24 g, 1.5 mol) was slowly added. After refluxing for 6 h, a yellow precipitate formed. After the product was allowed to stand at room temperature for half an hour, it was washed with saturated NaHSO_3_ solution to remove excess Br_2_. The mixture was filtered with suction, washed repeatedly with water, and dried to obtain the dibromo product as a gray solid. The yield was 90%. ^1^H NMR (400 MHz, chloroform-*d*) δ 7.73 (s, 2H).

#### 2.3.5. Synthesis of Polymers

The polymers were polymerized with the Stille coupling reaction. We chose P1 as an example to explain the synthesis steps.

EDOT (22 mmol, 2.4 mL) was dissolved in THF (70 mL) and cooled to −78 °C for 30 min. n-BuLi (2.5 M, 18 mL) was slowly added and reacted at −78 °C for 1 h then slowly increased to room temperature for 1 h. We cooled it down again to −78 °C, keeping it for 30 min, slowly dropped tributyltin chloride (45 mmol, 12.2 mL), slowly let it rise to room temperature, and stirred it overnight. The whole process requires N_2_ protection. After the reaction, the solution was washed with saturated NaHCO_3_ solution, the aqueous phase was extracted with petroleum ether, the organic phase was dried with anhydrous MgSO_4_, the organic phase was separated, and the solvent was spin-evaporated to obtain an oily liquid of 10.78 g with a yield of 68%.

M1 (1.28 g, 2 mmol) and 5,7-ditributyltin-2,3-dihydrothieno[3,4-b][1,4]dioxin (1.44 g, 2 mmol) were dissolved in THF (80 mL); then, we added Pd(PPh_3_)_4_ (6.96 mg, 0.006 mmol) and stirred it for ten minutes, reacted it at 66 °C for 24 h, and protected it with nitrogen throughout. After the reaction, the solution was poured into a large amount of methanol and stirred for 15 min. The solid was precipitated and filtered, and the solid was purified via Soxhlet extraction with methanol and hexane as solvents. It was further purified via column chromatography and dried in a vacuum to obtain a dark red solid of 0.89 g.

P2 was an orange powder solid, P3 was a yellow powder solid, and P4 was a purple-brown powder solid.

### 2.4. Preparation of the Polymer Films

The 10 mg polymer solid was dissolved in 3 mL DMF, then we carefully dripped it onto an ITO glass and dried it under a vacuum at 120 °C for 3 h to obtain the final polymer film.

## 3. Results and Discussion

### 3.1. FT-IR Spectra and TGA Curves

#### 3.1.1. FT-IR Spectra

**Figure 1:**
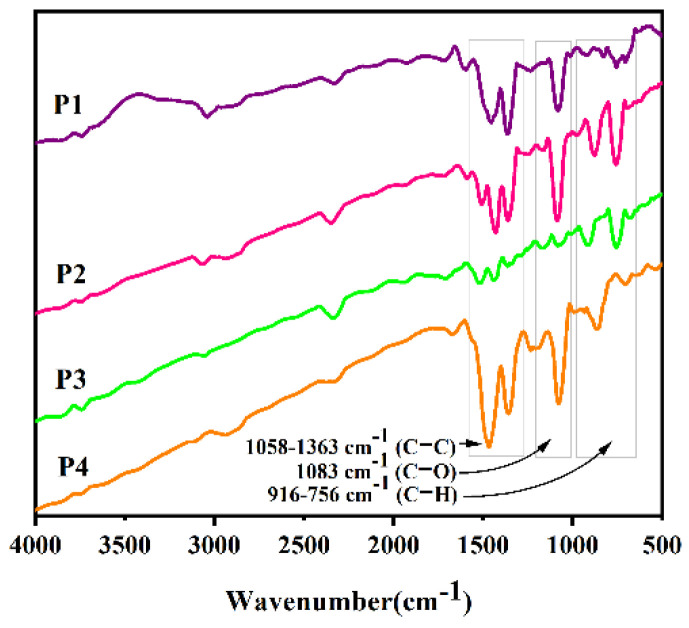
FT-IR spectra of the four polymers.

As shown in Figure 1, in order to further determine the structure of the polymer, an infrared analysis was performed on the four new types of polymers using infrared spectroscopy. Since a C-O bond does not exist in M1-M4 the structure but does exist in the EDOT structure, the key to successful polymerization is whether the absorption peak of the C-O bond can be observed in the infrared image. The C-O bond absorption peaks of P1–P4 are 1080 cm^−1^, 1082 cm^−1^, 1169–1080 cm^−1^, and 1080 cm^−1^, respectively, proving that four polymers were successfully synthesized.

#### 3.1.2. TGA Curves

**Figure 2:**
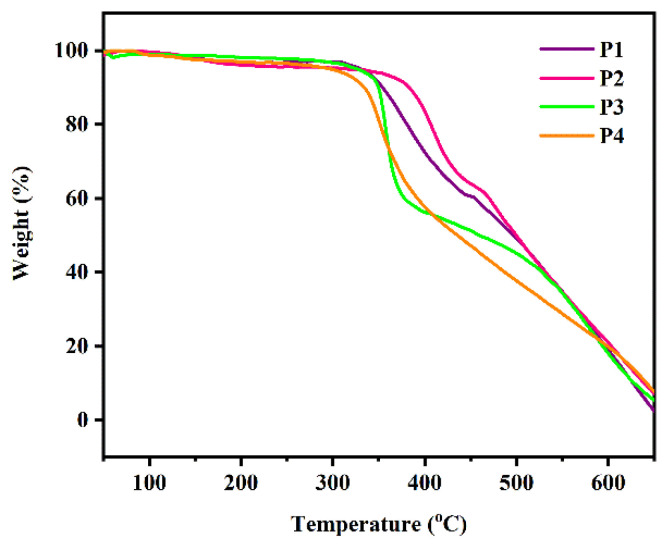
TGA curves of six polymers.

The thermal properties of P1–P4 were tested by thermogravimetric analysis at a heating rate of 10 °C·min^−1^ (Figure 2). Table 1 shows the decomposition temperatures of P1–P4 in air at 5%, 10%, and 20% and a char yield at 600 °C in air. At temperatures below 200 °C, no significant weight loss was observed in any polymers. These polymers have a weight loss temperature of about 350 °C at 10% in air. In addition, the residual carbonization (coke yield) of these polymers in air at 600 °C is 17–20 wt.%. Thermal analysis results showed that polymers P1–P4 exhibited similar thermal stability due to their similar structures. They are relatively stable before 300 °C and begin to decompose at 350 °C, indicating that these polymers have stable properties and a stable working time under normal conditions.

In addition, the molecular weight of the polymer was determined. In this experiment, the Viscotek-VE3580-RI-DETECTOR gel chromatograph produced by a British company called Malvern was used to test polymer molecular weight. The testing solvent used chromatographic pure DMF solvent, the testing temperature was constant at 35 °C, and the solvent flow speed during testing was 1.0 mL/min. The polydispersity coefficient (PDI) and weight-average molecular weight (Mw) of the polymer were about 1.4–1.9 and 4000–8400, respectively, and we found that the polymer contains about 7–9 structural units. Polymers with larger molecular weights cannot be completely dissolved in solvents, resulting in differences between the polydispersity coefficients of the polymers, and different polymer molecular weights can be attributed to differences in their degree of polymerization.

### 3.2. The Solubilities and AFM of the Polymers

Table 2 shows the solubility test results for the polymers. To do so, we weighed a 10 mg sample and dissolved it in 1 mL of solvent for a qualitative solubility test. From this table, we found that these polymers can be dissolved in the following solvents and have good solubility. In short, the good solubility of these polymers lends them greater potential in applications.

**Figure 3:**
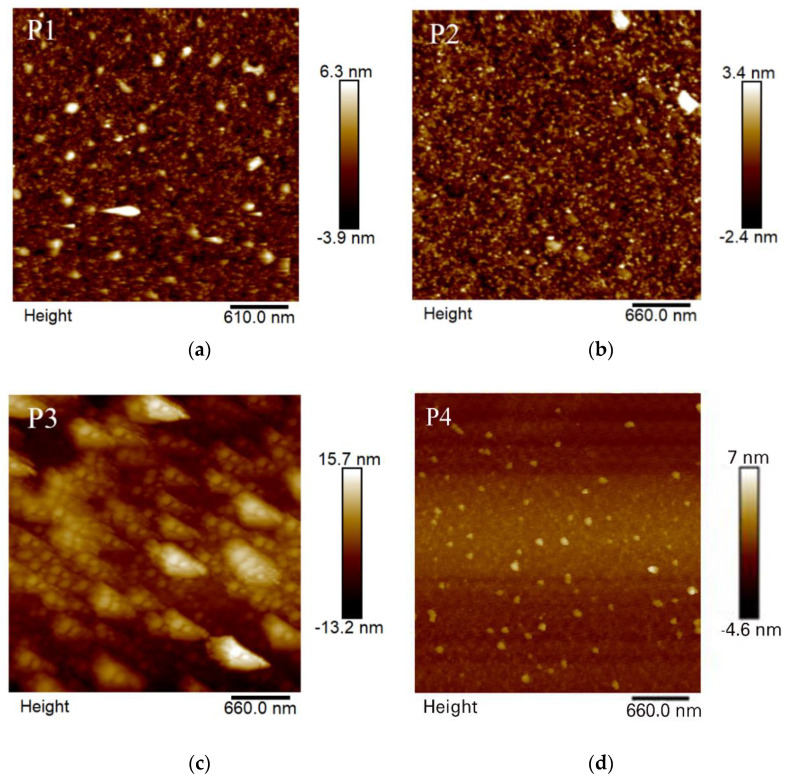
AFM images of polymer films. P1 (**a**), P2 (**b**), P3 (**c**), and P4 (**d**).

An analysis of the roughness of the polymer films using AFM is shown in Figure 3, where P1 and P2 have a smoother surface morphology than P3 and P4. The root means square roughness (Rq) of P1, P2, P3, and P4 was 1.30 nm, 0.81 nm, 4.10 nm, and 1.81 nm, respectively. The Rq of P2 is smaller and shows a smoother surface morphologically. The smaller the roughness of the surface, the smaller the probability of electrons colliding with the surface of the film, which leads to a increase in electrical conductivity and a decrease in the electrical resistivity of the film. Since the monomer and EDOT can freely rotate around the single bond during polymerization, which increases the uncertainty of aggregation, the roughness of the four polymers may change compared with the expected value.

### 3.3. Optical Properties

#### 3.3.1. UV–Vis Spectra

**Figure 4:**
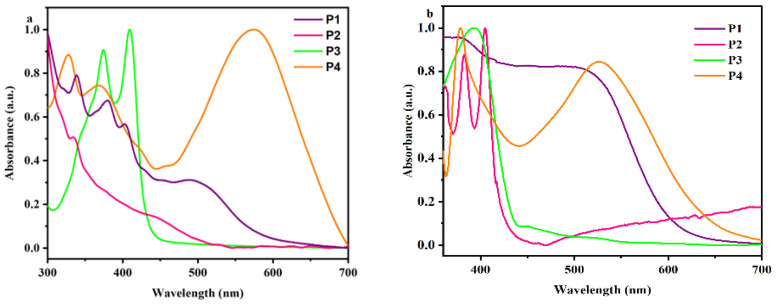
Absorption spectra of four polymers in DMF solution (**a**) and in film (**b**).

Figure 4 is the UV–vis spectra of the polymers. The absorption spectra of the four polymers in DMF solution (a) and in the film state (b) were tested. Table 3 is a summary of the UV–vis spectra of the polymers. P1 shows multiple absorption peaks at 345, 380, and 404 nm and an absorption peak at 507 nm. This is attributable to the existence of multiple conjugate paths in P1. An insignificant peak of P2 was found at 340 nm. P3 has two distinct absorption peaks at 375 nm and 409 nm. The maximum absorption wavelength of P4 is at 576 nm. The longest absorption peak is due to the conjugated main chain of the polymer, and multiple shortwave absorption peaks are due to the sidechain [26]. The UV-vis absorption spectra of the four polymers in the film state are as follows: P1 has a characteristic absorption peak at 520 nm, P2 has two absorption peaks at 385 nm and 405 nm, P3 has a characteristic absorption peak at 396 nm, and P4 has a characteristic absorption peak at 527 nm. The greater the maximum absorption wavelength of the polymer, the smaller the energy gap, and the higher the conjugation state of the electron cloud.

#### 3.3.2. PL Spectra

**Figure 5:**
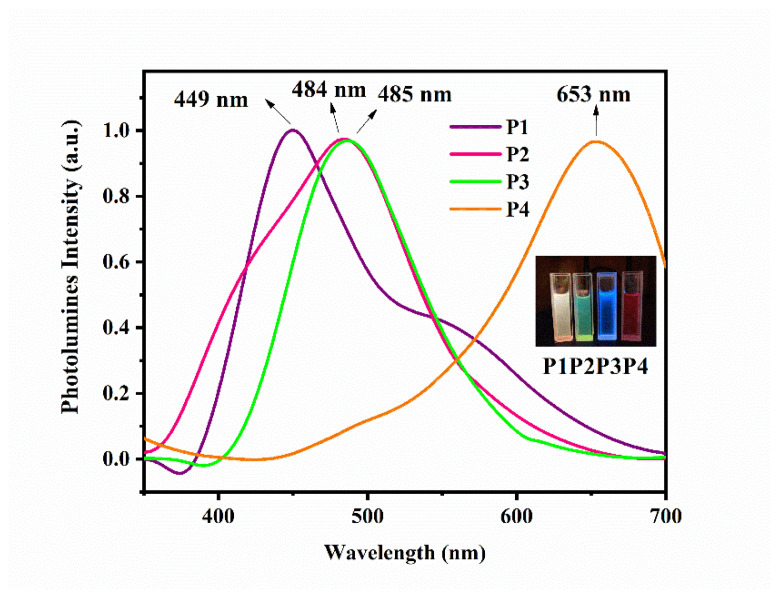
PL spectrum of polymers.

Due to the good solubility of the polymer in DMF and its high polarity, this will form an intramolecular charge transfer (ICT) state, which will reduce the polymer’s emission energy state. This will affect the fluorescence test. As shown in Figure 5, the maximum emission wavelength range of the PL spectra of P1, P2, P3, and P4 in THF is 449–653 nm. The P1–P4 solution shows white, green, blue, and red fluorescence, respectively. The strong fluorescence of the solution indicates that the introduced large conjugate aromatic group can effectively inhibit the twisted intramolecular charge transfer (TICT) state. The introduction of electron donor–acceptor (D-A) structures to molecules can improve the equilibrium injection and the transportation of electron or hole pairs, thus regulating the spectral properties of substances. Due to the electron-deficient nature of benzothiadiazole group, the electron-withdrawing group on the conjugated backbone pushes the optical absorption of the polymer to a significant redshift, so P4 shows the largest redshift feature in the spectrum.

### 3.4. Electrochemical Properties

We tested the electrochromic properties of four polymers using cyclic voltammetry (CV) as usual. When a scanning potential coincides with the oxidation or reduction potential of a polymer, the polymer generates an electric current by losing or gaining electrons. Therefore, to characterize the redox process of polymers, we only need to record the curve of current as a function of potential.

**Figure 6:**
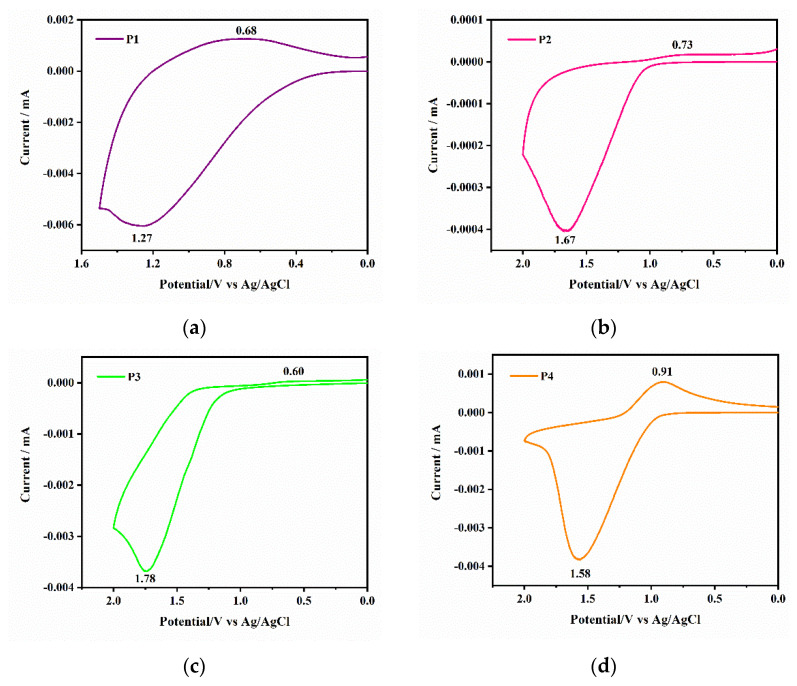
CV curves of P1–P4 on an ITO/glass electrode in 0.2 M TBAP/MeCN solution at a scan rate of 50 mV·s^−1^. P1 (**a**), P2 (**b**), P3 (**c**), and P4 (**d**).

As shown in Figure 6, the CV plot of P1 shows an oxidation peak at 1.27 V. According to previous reports, the oxidation peak comes from the formation of radical cations [27]. The initial oxidation potential of the polymer was tested by cyclic voltammogram. Due to the substitution of different types of aromatic rings, the initial oxidation potential of some of the polymers is 1.27 V, 1.67 V, 1.78 V, and 1.58 V, respectively. All polymers show complete redox peaks; the color change in the film is achieved through the redox process of the polymer. Polymer P1 exhibited the lowest initiation potential, and the oxidation potentials of P2 and P3 continued to increase. This is because the length of the monomer-conjugated chain increased, and the electron migration efficiency in the polymer chain continued to increase. This large aromatic-conjugated structure introduced into the polymer can effectively reduce the potential of free radical cations.

**Figure 7:**
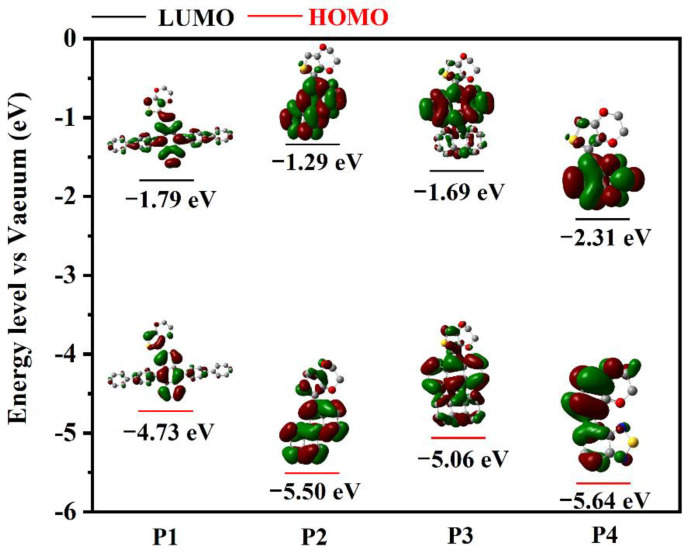
Pictorial representations of the electron density in the frontier molecular orbitals of P1–P4.

The dimer was used as the repeating unit in the DFT calculation. Table 4 summarizes some data on the HOMO and LUMO levels of the polymers. Figure 7 shows that the electron clouds of the polymer, HOMO and LUMO, are distributed in different positions. Because P4 contains a D-A structure, this structure can effectively enhance the electron transport capacity of the molecule, thus changing the energy gap of the substance and reducing the HOMO and LUMO levels of the polymer. P1 has the lowest initial oxidation voltage and the lowest theoretical energy band gap ($E_{g}^{\mathrm{quantun}}$); this is because P1 has more benzene rings than the other three monomers, which makes its conjugate structure larger, which also means that the starting voltage and energy consumption of the P1 device are relatively low, and it can have a wider range of applications.

### 3.5. Electrochromic and Spectroelectrochemical Properties of the P1–P4 Films

The spectroelectrochemical properties of the P1–P4 films were tested using the change in UV-vis absorption under the applied potential. We dissolved the polymer in an organic solvent and applied it to ITO-conductive glass using the drop-coating method. In order to study the electrochromic properties of polymer films at different voltages, UV-vis spectroscopy was used.

**Figure 8:**
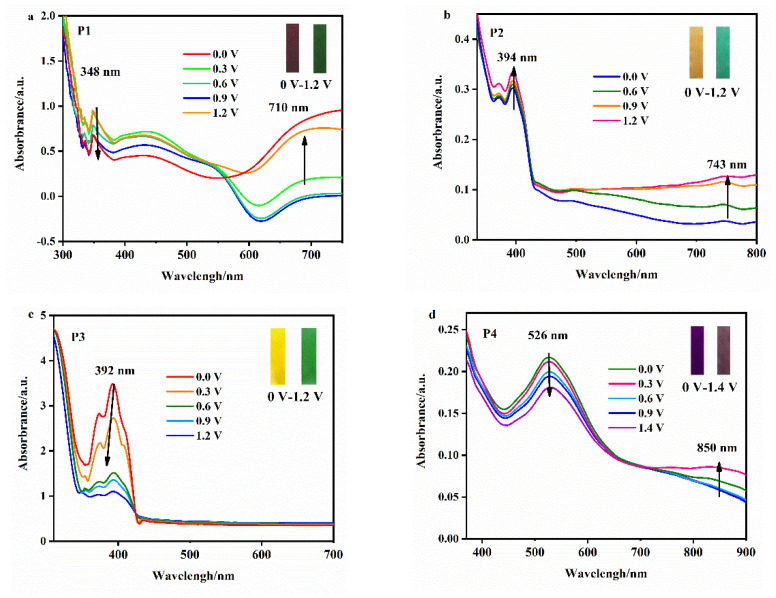
Electronic absorption spectra of the polymer films P1 (**a**), P2 (**b**), P3 (**c**), and P4 (**d**) in 0.2 M TBAP/CH_3_CN solution with different voltages.

As can be seen in Figure 8, when the voltage in the control system is 0–1.2 V, the internal properties of the polymer film change from a neutral state to an oxidation state, which manifests as a color change and a new absorption peak in the absorption spectrum. The P1 film changes from reddish-brown to green, with peaks at 348 nm and 710 nm. The P2 film changes from orange to cyan, with peaks at 394 nm and 743 nm. The P3 film changes from yellow to yellow-green. Since the absorption wavelengths of the two colors are similar, only the blue shift of the absorption peak occurs after the voltage increases, and there is no obvious new absorption peak. When the control voltage is between 0 V and 1.4 V, the P4 film changes from purple to brown, with peaks at 526 nm and 850 nm. The above phenomena may be due to the fact that the structure of the EDOT unit and aromatic ring unit are connected, resulting in the enhancement of the conjugation degree and the adjustment of the structure, and electron transfer under the action of voltage causes the color change.

### 3.6. Electrochromic Switching

In order to further evaluate the electrochromic properties of the polymers, we studied the coloring efficiency (CE) and response time of the polymers and monitored the change in the transmittance (ΔT) of the polymers for a period during their maximum absorption.

The coloration efficiency (CE) can be calculated using the related equations provided below:

(1) $$\Delta \mathrm{OD}=\mathrm{lg}T_{b}/T_{c}$$

(2) $$\mathrm{CE}=\Delta \mathrm{OD}/Q_{d}$$

In the formula, Q_d_ is the number of ejected charges determined experimentally, T_c_ is the transmittance in the colored states, and T_b_ is the transmittance in the bleached states.

The reaction time was one of the most important properties of the electric color change. It was defined as the time needed to reach 90% of the change in the maximum transmission when the electric potential changed [28,29].

**Figure 9:**
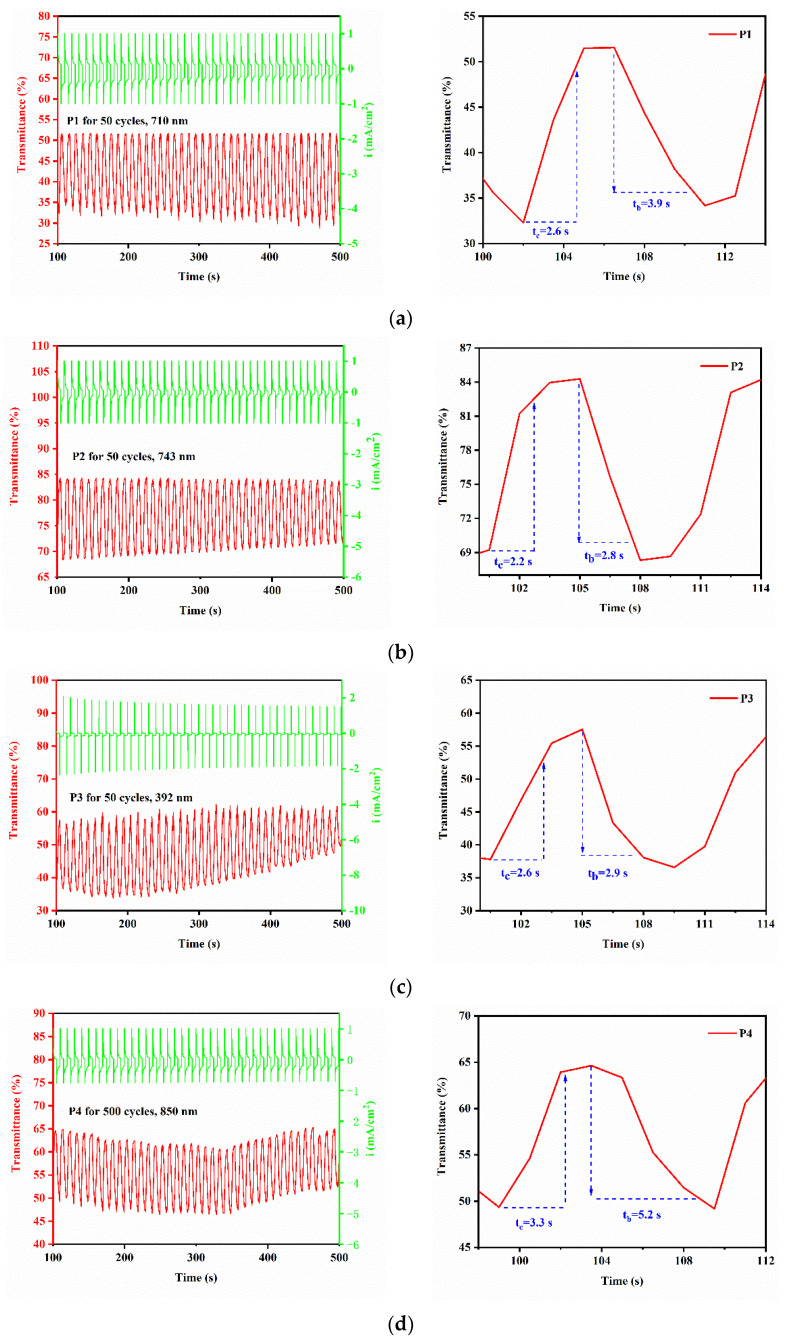
Dynamic changes in the current and transmittance and switching time for P1–P4 (in 0.2 mol·L^−1^ TBAP/CH_3_CN). P1 (**a**), P2 (**b**), P3 (**c**), and P4 (**d**).

From Figure 9, we can see that the incident wavelengths of the P1–P4 films are 710 nm, 743 nm, 392 nm, and 526 nm, respectively. Taking the P1 film as an example, at 710 nm, the coloring time is 2.6 s, the bleaching time is 3.9 s, and the bleaching time is 1.3 s slower than the coloring time. The time difference occurs because the internal resistance of the polymer is high and because the structure makes it relatively difficult for electrons to transport in the conjugated conductive environment. In addition, the coloring times of P2, P3, and P4 are 2.2 s, 2.6 s, and 3.3 s, respectively, and the bleaching times are 2.8 s, 2.9 s, and 5.2 s, respectively. By comparing the four polymers in this group with each other, P2 has a shorter response time.

Table 5 shows that the coloring efficiencies of P1–P4 are 105.3 cm^2^·C^−1^, 47.4 cm^2^·C^−1^, 283.3 cm^2^·C^−1^, and 84.6 cm^2^·C^−1^, respectively. Among them, the coloring efficiency of P3 is the highest, which means that P3 can change color more easily.

We can find from the figure that the polymer film has relatively good cycle stability within 50 cycles.

### 3.7. Storage Mechanism of the Devices

#### 3.7.1. Preparation of Resistance-Switching Memory Device

**Figure 10:**
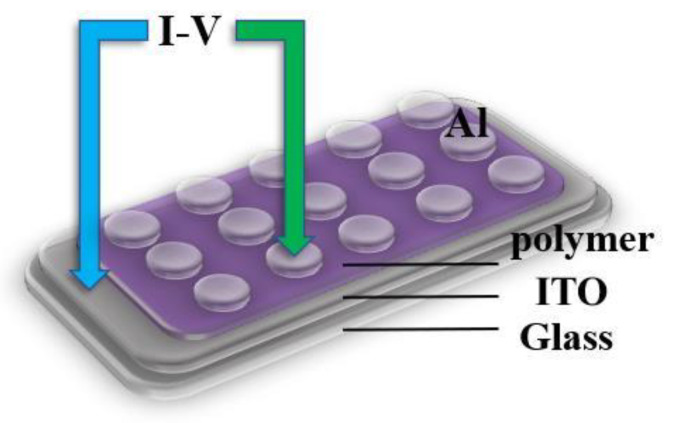
Schematic diagram of multilayer resistive switching.

The 2 cm × 1 cm ITO glass was ultrasonically cleaned for 20 min with water, acetone, and ethanol, in that order. We dissolved 3 mg of polymer in 1 mL of DMF, and the solution was dropped on the conductive surface of the ITO with an eyedropper. Then, it was placed flat in a vacuum oven at 90 °C until the solvent was completely baked dry [30]. Finally, a 300 nm thick Al electrode top layer was deposited by thermal deposition and evaporated through a shadow mask at a pressure of 1.0 × 10^−4^ Pa to obtain an ITO/polymer/Al sandwich device (as shown in Figure 10).

#### 3.7.2. Analysis of Storage Performance of Resistance-Switching Storage Devices

Current–voltage (I-V) tests were performed to characterize the performance of the ORRAM devices. The resistive memory device consisted of a simple sandwich structure in which the polymer could be used as the active layer, Al as the top electrode (this is because Al can be deposited on the device with vacuum evaporation), and the ITO as the bottom electrode [31].

**Figure 11:**
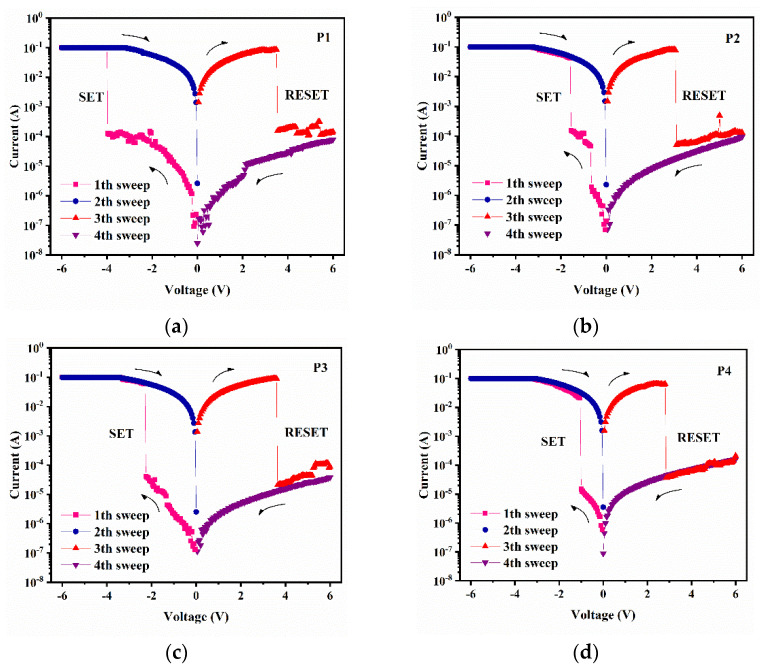
Typical I-V characteristics of the ITO/polymer/Al memory devices: P1 (**a**), P2 (**b**), P3 (**c**), and P4 (**d**).

**Figure 12:**
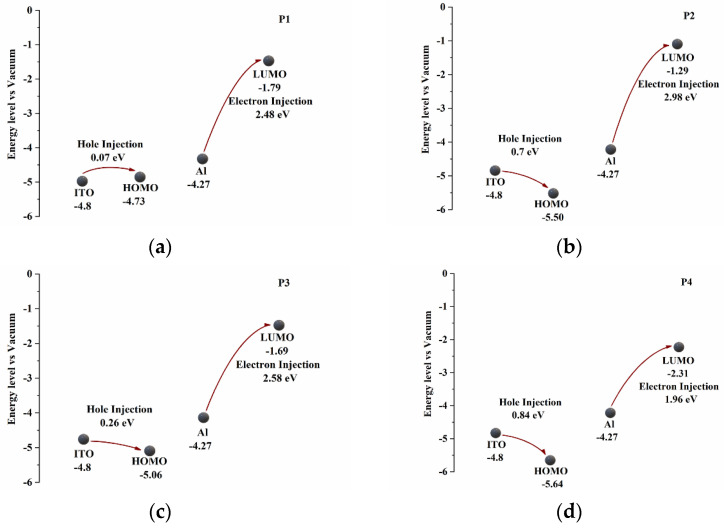
Resistive memory device energy level diagram of the polymers. P1 (**a**), P2 (**b**), P3 (**c**), and P4 (**d**).

As shown in Figure 11 and Figure 12, the I-V characteristic curve consists of four-step scanning with varying voltage applied. Take P1 for example. First, as the voltage changes from 0 V to 6 V, the current starts to increase slowly. The current suddenly increases at 3.9 V, a process we call writing, and the high resistance state (HRS) of the device is converted to a low resistance state (LRS), and the voltage is called the threshold voltage. The on/off current ratio of P1 during conversion is 796.83. In the second step, the voltage changes; this is the “read” step. Next, a reverse voltage is applied to the device, and when the voltage reaches 3.5 V, the device suddenly returns to the HRS state, which is another threshold voltage, which is the memory-erasing process. In the fourth step, another 6 to 0 V of voltage is applied, and the device displays the low-resistance state (LRS), which is a “reread” process. These two processes of sharp increase and decrease in current are called “set” and “reset”. In addition, the rewritable electrical characteristics generated by the four-step scan make up the entire “write–read–erase–reread” cycle (WRER). Both settings and resets show nonvolatile characteristics, indicating that this is a typical flash memory type.

Similar to the P1-based devices, the P2–P4-based devices have threshold voltages of 1.5 V, 2.3 V, and 0.9 V, respectively, and on/off current ratios of 296.00, 1582.80, and 2527.42, respectively (as shown in Table 6). To evaluate the performance of a memory device, the following points are generally required: 1. The change in the conduction state has a larger on/off current ratio; that is, the device has a lower misread rate. 2. In a shorter time, a bistable transition process can occur. 3. The transition process has a lower threshold voltage, usually less than 6 V. 4. It can maintain a stable working state at room temperature. These are all decisive factors to judge whether a device has high-performance development and application [32,33,34,35]. Therefore, the storage performance of P4 is much higher than that of P1, P2, and P3, and we believe that the device has excellent nonvolatile, bipolar resistance variable storage characteristics.

## 4. Conclusions

In short, we successfully synthesized a series of new polymers with EDOT units through the Stille coupling reaction. Because all the polymers contain large conjugated groups, they have excellent electron transportation capabilities and perform well in electrochromic conditions. Among them, P3 shows a higher coloring efficiency (283.3 cm^2^·C^−1^), and P2 shows a shorter response time (t_c_ = 2.2 s, t_b_ = 2.8 s). In addition, these polymers all have the property of resistive-switching storage; in particular, the switching current ratio of P4 is as high as 2527, and the threshold voltage is as low as 0.9 V. Based on the above results, our work provides a favorable idea for electrochromic and resistive switch storage materials, and these novel multifunctional polymers have a great future in optoelectronic functional materials.

## Figures and Tables

**Table 1:** Thermal properties of the polymers.

Polymer Code	T_5%_ ^a^	T_10%_ ^a^	T_20%_ ^a^	Char Yield ^b^ (wt.%)	M_w_	M_n_	PDI
P1	267	330	380	17.8	8400	5800	1.4
P2	207	384	407	19.5	5200	2700	1.9
P3	320	350	357	16.5	6700	3800	1.7
P4	299	335	351	19.6	4000	2200	1.8

^a^ Decomposition temperatures (T_d_) at 5%, 10%, and 20% weight loss in air. ^b^ Measured at 600 °C in air.

**Table 2:** Solubility of P1–P4 in organic solvents.

Polymer Code	Solubility in Various Solvents ^a,b^					
	DMF	DMAC	DMSO	THF	CH_3_CN	Toluene
P1	+ +	+ +	+ +	− −	− −	− −
P2	+ −	+ +	+ +	+ −	− −	− −
P3	+ +	+ +	+ +	+ −	− −	− −
P4	+ −	+ −	+ +	− −	− −	− −

^a^ Solvents: CH_3_CN: acetonitrile; DMAc: N, N-dimethylacetamide; DMSO: dimethyl sulfoxide; THF: tetrahydrofuran; NMP: N-methyl-2-pyrrolidone. ^b^ The qualitative solubility of the polymer was measured with 10 mg of specimen in 1 mL of solvent with stirring. + +: soluble under room temperature; + −: partly soluble under hot temperature; − −: insoluble even when heated.

**Table 3:** Optical properties of the polymers.

Polymers	In Solution ^a^ λ_max_ (nm)	as Film λ_max_ (nm)
P1	507	520
P2	340	405
P3	409	396
P4	576	527

^a^ The concentration measured in DMF is 1 × 10^−5^ mol·L^−1^.

**Table 4:** Optical and electrochemical properties of the polymers.

Polymer Code	$E_{\mathrm{onset}}^{\mathrm{OX}}$ (V)	E_HOMO_ ^a^ (eV)	E_LUMO_ ^b^ (eV)	E_g_ ^c^ (eV)	λ_onset_ ^d^ (nm)	$E_{HOMO}^{quantun}$ (eV)	$E_{LOMO}^{quantun}$ (eV)	$E_{g}^{quantun}$ (eV)
P1	0.41	−4.84	−2.69	2.15	576	−4.73	−1.79	2.94
P2	1.09	−5.52	−2.79	2.73	455	−5.50	−1.29	4.21
P3	1.20	−5.63	−2.82	2.81	442	−5.06	−1.69	3.37
P4	1.03	−5.46	−3.57	1.89	657	−5.64	−2.31	3.33

^a^ E_HOMO_ = −e ($E_{\mathrm{onset}}^{\mathrm{OX}}$ vs. Ag/AgCl + 4.43) eV. ^b^ E_LUMO_ = E_HOMO_ + E_g_. ^c^ E_g_ = 1240/λ_onset_. ^d^ UV-vis absorption onset of the polymers in DMF. E_quantum_: theoretical calculation of the polymers.

**Table 5:** Electrochromic properties of the polyamide films.

Polymer Code	λ_max_^a^ (nm)	Δ%T	t_b_ ^b^ (s)	t_c_ ^b^ (s)	ΔOD ^c^	Q_d_ ^d^ (mC·cm^−2^)	CE ^e^ (cm^2^·C^−1^)
P1	710	20.2	3.9	2.6	0.20	1.9	105.3
P2	743	15.1	2.8	2.2	0.09	1.9	47.4
P3	392	19.7	2.9	2.6	0.17	0.6	283.3
P4	850	14.6	5.2	3.3	0.11	1.3	84.6

^a^ Maximum absorption wavelength of P1–P4. ^b^ Time for 90% of the full-transmittance to change. ^c^ Optical density (ΔOD) = log [T_bleached_/T_colored_]. T_bleached_ is the maximum transmittance in the neutral states and T_colored_ is the maximum transmittance in the oxidized states. ^d^ Q_d_ is an ejection charge that was tested by experiments. ^e^ Coloration efficiency (CE) = ΔOD/Q_d_.

**Table 6:** Resistance-switching memory characteristics of the polymers.

Polymers	On/Off Current Ratios	Threshold Voltages (V)
P1	796.83	3.9
P2	296.00	1.5
P3	1582.80	2.3
P4	2527.42	0.9

## Data Availability

The data that support the findings of this study are available from the corresponding author upon reasonable request.

## Supplementary Materials

The following supporting information can be downloaded at: https://www.mdpi.com/article/10.3390/polym14224965/s1, Figure S1: ^1^H NMR spectrum of M1. Figure S2: ^1^H NMR spectrum of M2. Figure S3: ^1^H NMR spectrum of M3. Figure S4: ^1^H NMR spectrum of M4. Figure S5: ^1^H NMR spectrum of P1. Figure S6: ^1^H NMR spectrum of P2. Figure S7: ^1^H NMR spectrum of P3. Figure S8: ^1^H NMR spectrum of P4. Figure S9: ^13^C NMR spectrum of P1 (CDCl_3_). Figure S10: ^13^C NMR spectrum of P3 (CDCl_3_).
