# Supplementary material for: Conjugated Polymers Containing EDOT Units as Novel Materials for Electrochromic and Resistance Memory Devices
Source: Polymers (Basel). 2022 Nov 16;14(22):4965. doi: 10.3390/polym14224965 (PMC9696808; doi:10.3390/polym14224965)
Supplement: Supplementary file 1 [file polymers-14-04965-s001.zip › polymers-1963894-supplementary.pdf]

Supplementary Material

Conjugated Polymers Containing EDOT Units as Novel Materials for Electrochromic and Resistance Memory Devices

Zipeng He [1], Haoran Xu [1], Yuhang Zhang [1], Yanjun Hou [1,*] and Haijun Niu [2,*]

[1] Key Laboratory of Chemical Engineering Process and Technology for High-Efficiency Conversion, College of Heilongjiang Province, Heilongjiang University, Harbin 150080, China

[2] Key Laboratory of Functional Inorganic Material Chemistry, Ministry of Education of the People's Republic of China, Heilongjiang University, Harbin 150080, China

[*] Correspondence: houyj@hlju.edu.cn (Y.H.); haijunniu@hotmail.com (H.N.)

^{1}H NMR and ^{13}C NMR

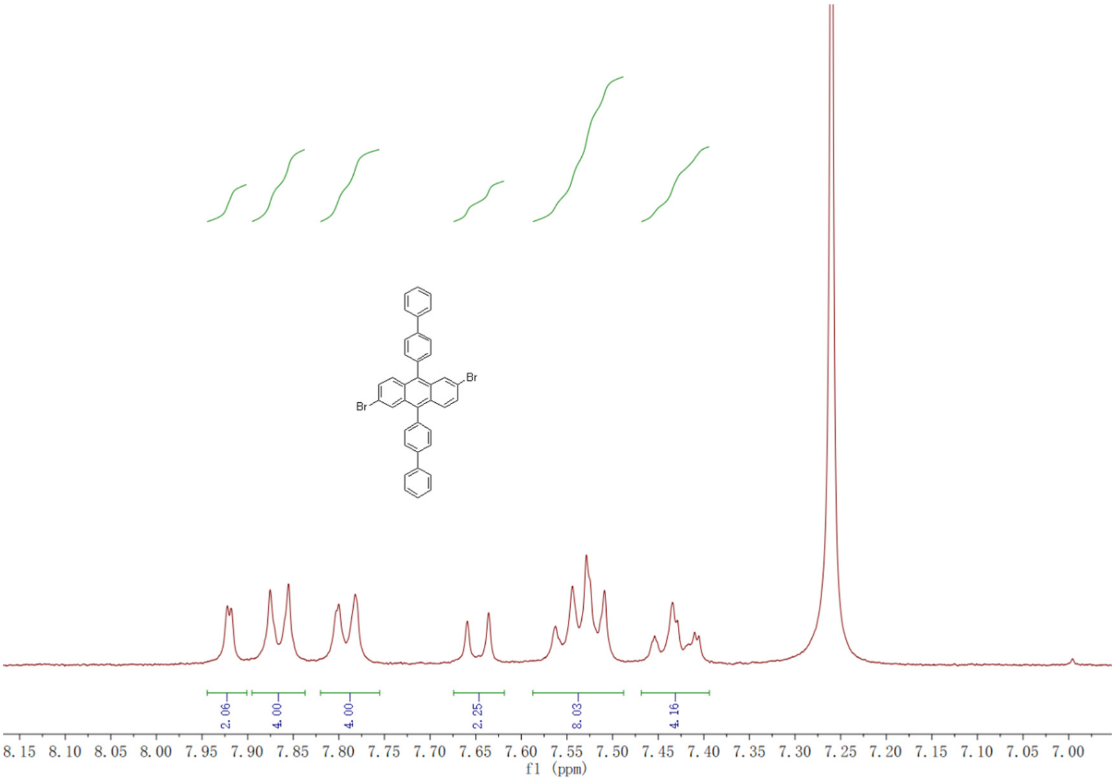

Figure S1. ^{1}H NMR spectrum of M1.

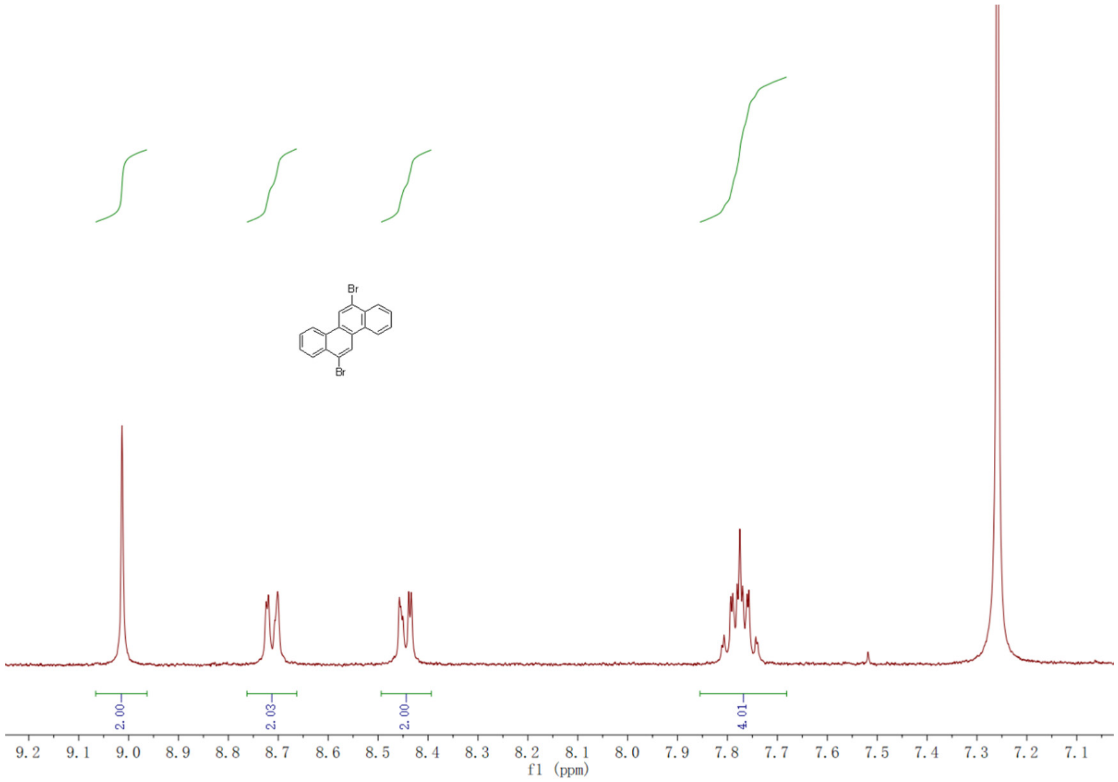

Figure S2. ^{1}H NMR spectrum of M2.

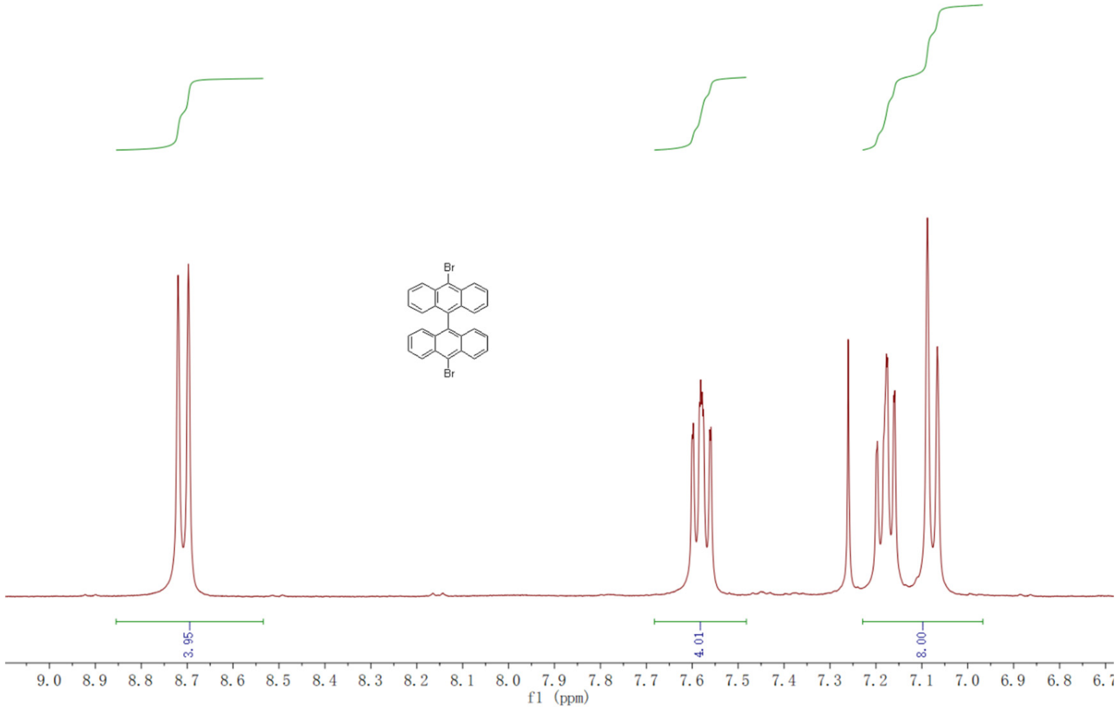

Figure S3. ^{1}H NMR spectrum of M3.

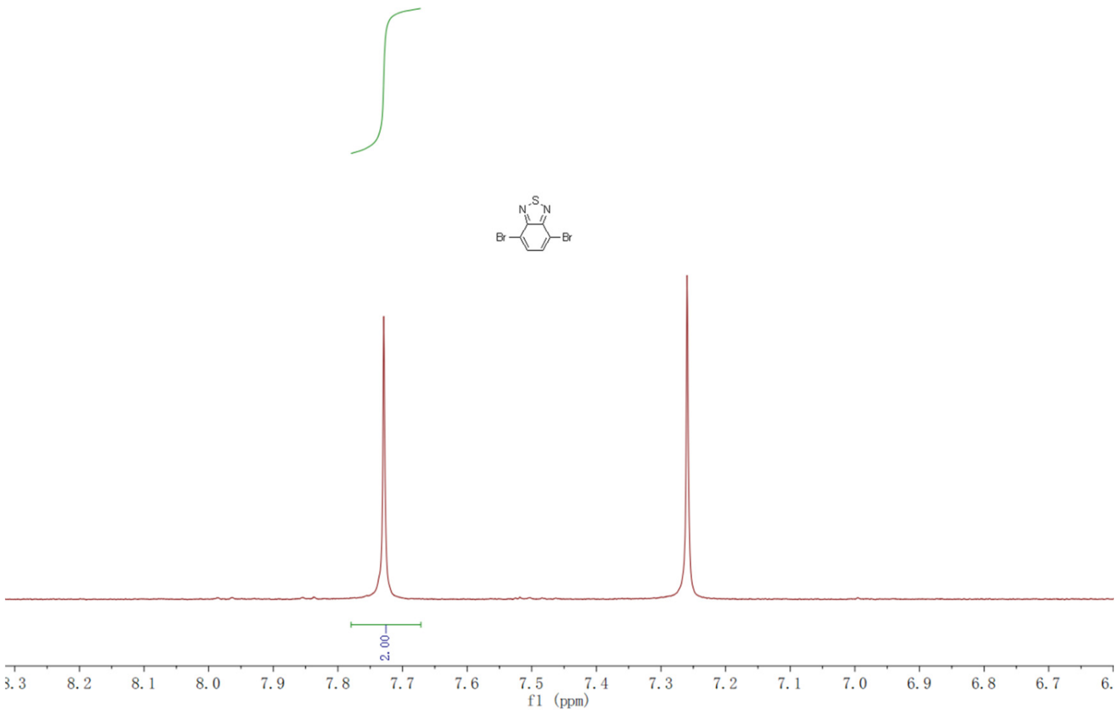

Figure S4. ^{1}H NMR spectrum of M4.

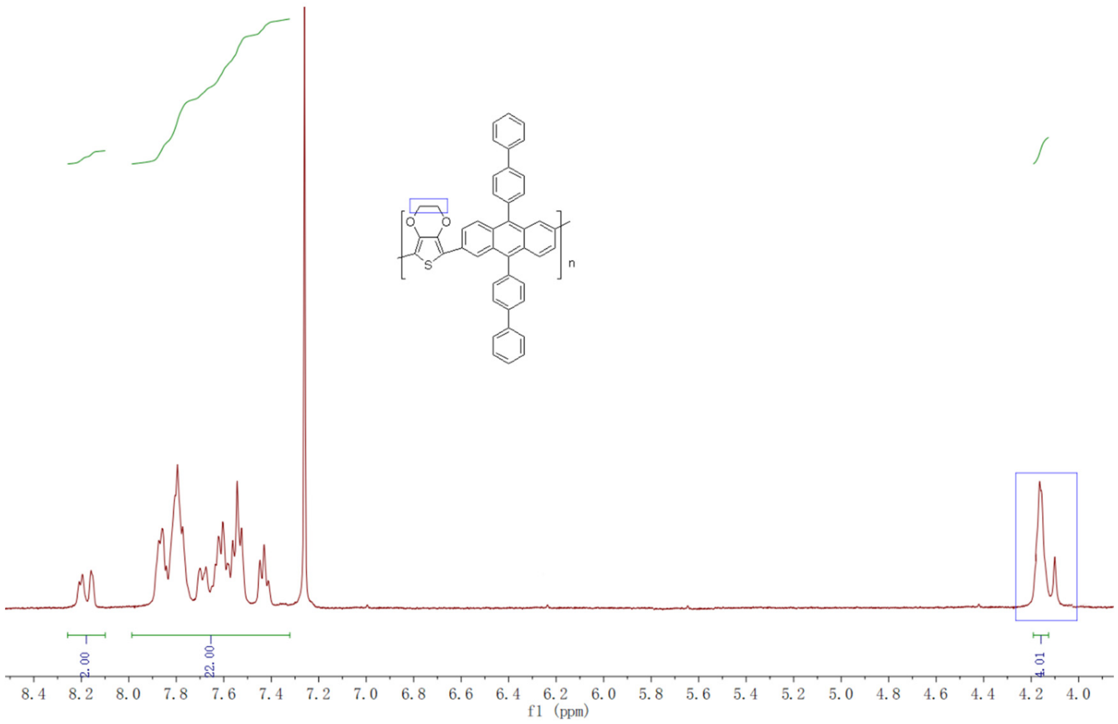

Figure S5. ^{1}H NMR spectrum of P1.

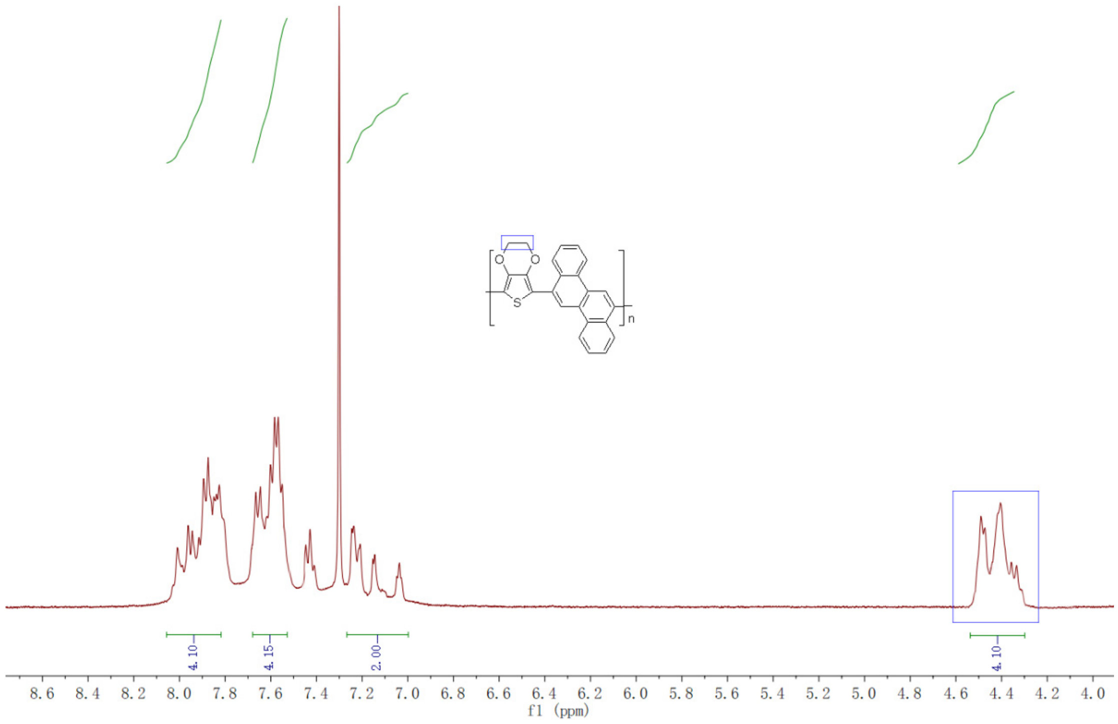

Figure S6. ^{1}H NMR spectrum of P2.

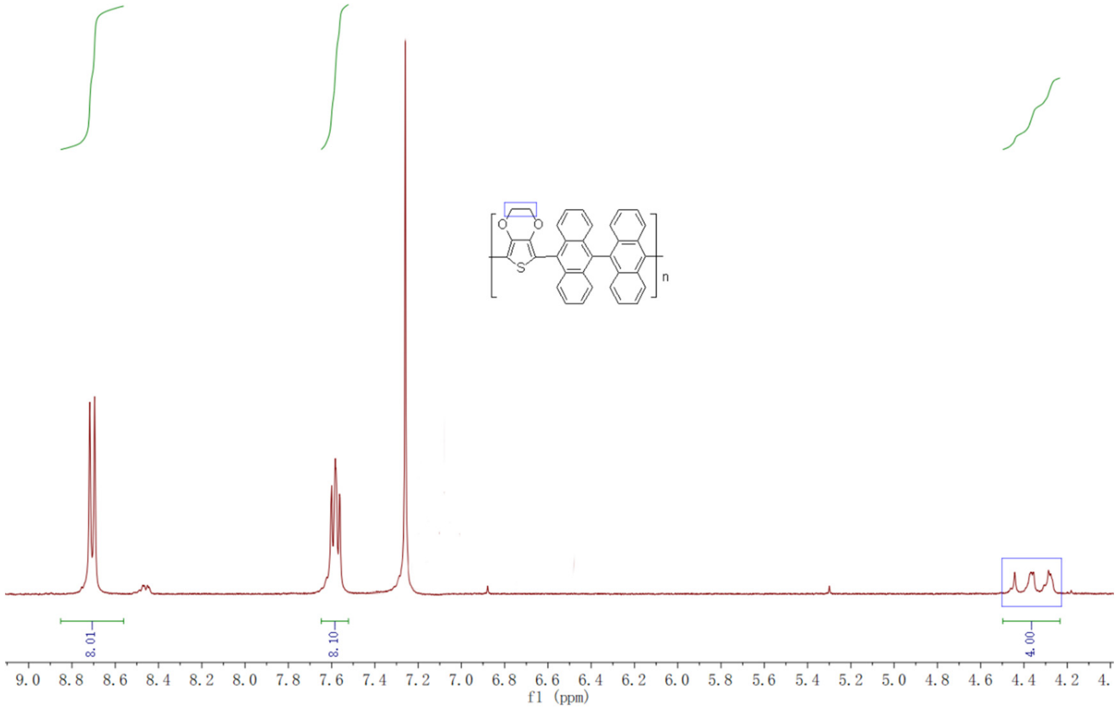

Figure S7. ^{1}H NMR spectrum of P3.

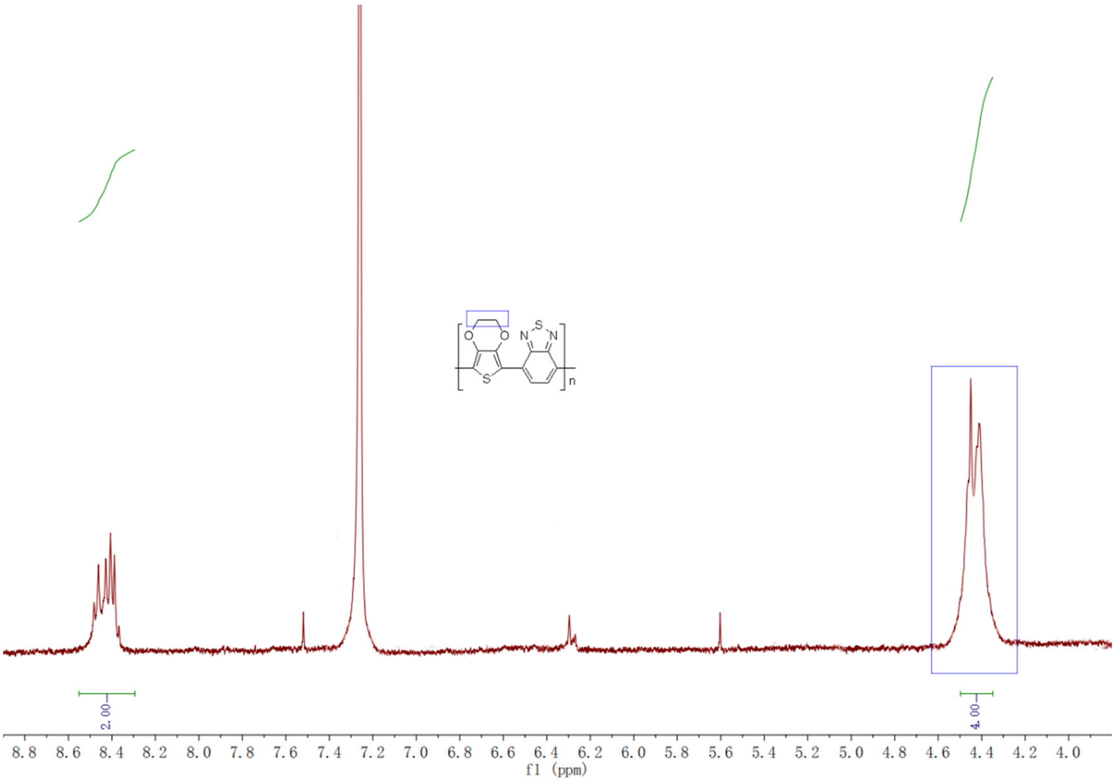

Figure S8. ^{1}H NMR spectrum of P4.

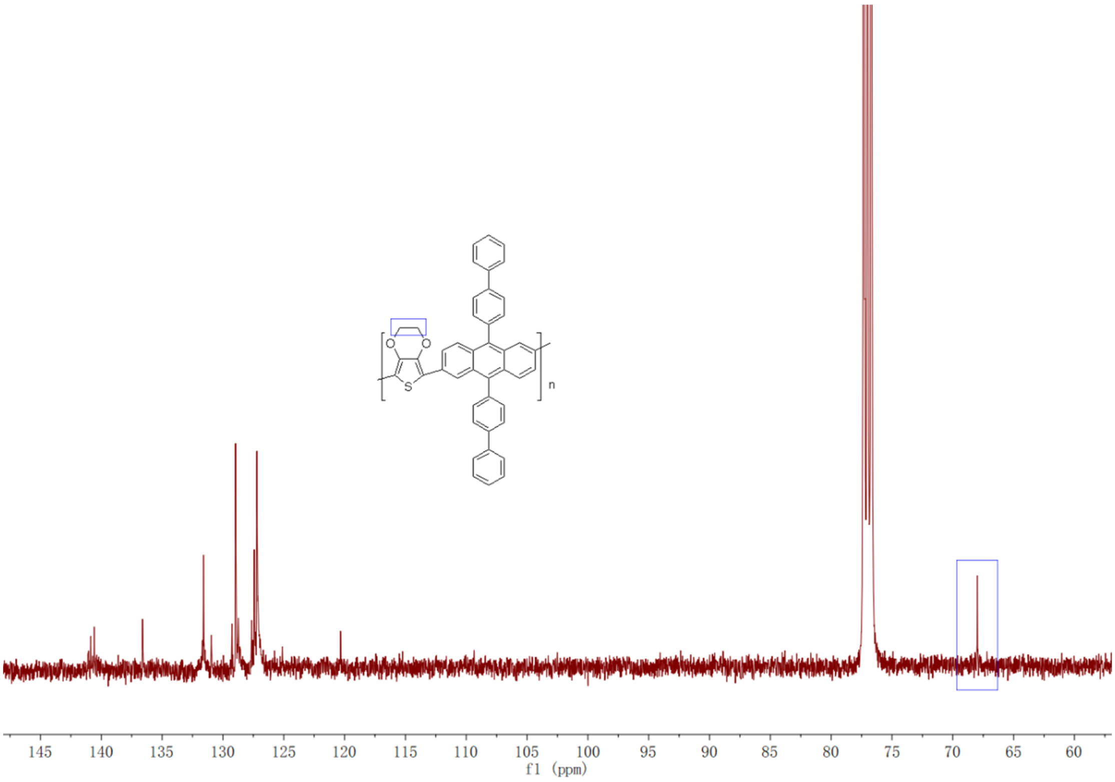

Figure S9. ^{13}C NMR spectrum of P1. ($CDCl_3$)

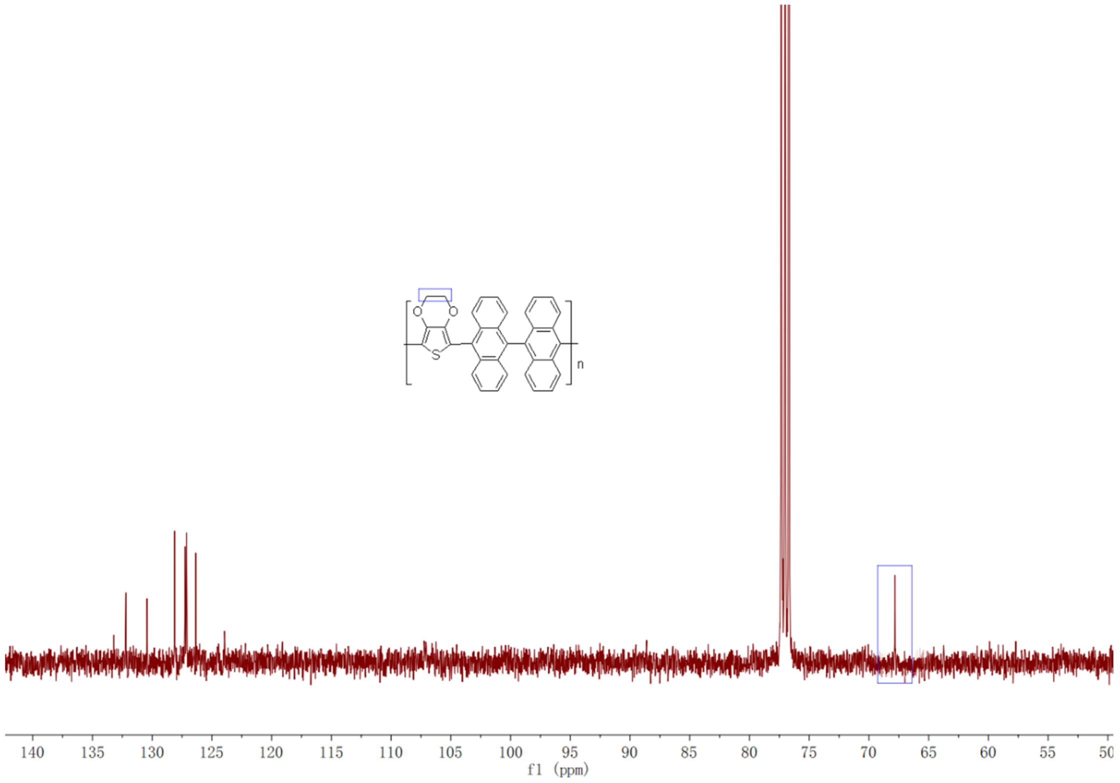

Figure S10. ^{13}C NMR spectrum of P3. ($CDCl_3$)

Unfortunately, due to solubility problems in $CDCl_3$ and DMSO-d_6, the ^{13}C NMR spectra of P2 and P4 did not be obtained.
